# Maternal, Embryofetal and Teratogenic Toxicity of Gel-Forming Galactomannan from *Cassia grandis* in Wistar Rats

**DOI:** 10.3390/gels12100936

**Published:** 2026-10-18

**Authors:** Daniel Medeiros Nunes, André Luís Salvino Alves de Oliveira, Alisson Rodrigo da Silva Oliveira, Anísio Francisco Soares, Teresinha Gonçalves da Silva, Luiza Rayanna Amorim de Lima, Natalie Emanuelle Ribeiro Rodrigues, Priscilla Barbosa Sales de Albuquerque

**Affiliations:** 1 Department of Medicine, University of Pernambuco (UPE), Garanhuns Campus, Garanhuns 53.294-902, PE, Brazil; daniel.medeirosnunes@upe.br (D.M.N.); andre.salvino@upe.br (A.L.S.A.d.O.); luiza.amorim@upe.br (L.R.A.d.L.); natalie.rodrigues@upe.br (N.E.R.R.); 2 Laboratory of Farmatoxicological Prospecting of Bioactive Products (BIOFARMATOX), Department of Antibiotics, Federal University of Pernambuco (UFPE), Recife Campus, Recife 54.740-520, PE, Brazil; alissonrodrigoo@hotmail.com (A.R.d.S.O.); teresinha.goncalves@ufpe.br (T.G.d.S.); 3 Department of Morphology and Animal Physiology, Animal Bioscience Program, Federal Rural University of Pernambuco (UFRPE), Recife Campus, Recife 52.171-900, PE, Brazil; anisio.soares@ufrpe.br; 4 Institute of Biological Sciences (ICB), University of Pernambuco (UPE), Recife Campus, Recife 50.100-130, PE, Brazil

**Keywords:** gel-forming polymer, reproductive toxicity, polysaccharide, embryotoxic, organogenesis, teratogenic

## Abstract

This study aimed to evaluate the potential maternal and developmental toxicity of orally administered galactomannan extracted from *Cassia grandis* seeds during the pre-implantation and organogenesis periods in Wistar rats. Considering the promising biotechnological potential of this gel-forming polysaccharide, its safety evaluation during pregnancy is essential. In vitro analysis using the MTT assay demonstrated that galactomannan (1–10 µg/mL) was not cytotoxic to L929 fibroblasts. In vivo, pregnant rats treated with 0.1, 1.0, or 10 mg/kg showed no clinical signs of maternal toxicity, including alterations in behavior, body weight gain, or food or water intake. Additionally, no alterations were observed in the absolute or relative weights of maternal organs. During the pre-implantation phase, no differences were observed in the number of corpora lutea, implantation sites, or pre-implantation losses. Histological analyses of implantation sites and placentas revealed normal morphology in all groups. Exposure during organogenesis did not induce external, visceral, or skeletal malformations, and no significant changes were detected in reproductive parameters. A significant overall difference in fetal body weight was observed with a significant pairwise difference only between the 0.1 and 10 mg/kg groups; however, neither group differed significantly from the control. In conclusion, the oral administration of *C. grandis* galactomannan at the tested doses did not produce maternal, embryotoxic, or teratogenic effects in Wistar rats. However, further studies involving broader dose ranges, larger sample sizes, and formulation-specific assessments are necessary before conclusions regarding reproductive safety in biomedical applications can be established.

## 1. Introduction

The use of natural or synthetic polymers has been growing in different industries, from food to pharmaceutical ones [1]. Polysaccharides are considered one of the main representatives from the group of natural polymers; they are complex carbohydrates extracted from plants, animals, fungi, and algae, or obtained via fermentation [2,3], and the focus in many studies due to their important properties, such as biodegradability, biocompatibility, low cost of extraction, and low or nontoxicity [4]. More specifically, polysaccharides derived from plants are part of the history of herbal ingredients and have been the most exploited due to their benefits to human health [2].

*Cassia grandis* Linn is a small- or medium-sized tree belonging to the Caesalpiniaceae family and native to Central and Tropical America and the West Indies [5]. It is commonly found as an ornamental tree in gardens and avenues with dicotyledonous, brownish, oval, medium-sized seeds containing about 50% endosperm gum, which possess the characteristics of becoming a potential source of seed gum. The purified polysaccharide from seeds collected in India was characterized by Joshi and Kapoor (2003) [6] as a pure galactomannan with a mannose–galactose ratio of 3.15 and a β-(1→4)-linked main mannan backbone to which galactose units are attached at O-6. The galactomannan obtained from the seeds of *C. grandis* collected in the northeast of Brazil was reported to have a similar structure composed of a central core of 4-linked β-D-mannose units with branches of D-galactose linked to the carbohydrate core through an α-(1–6) linkage; in this case, the mannose–galactose ratio was 2.44 [7], and the degree of crystallinity found by X-ray diffraction was 0.334.

Previous characterization demonstrated that increasing galactomannan concentration results in increased viscosity and the development of viscoelastic behavior, with a reported sol–gel transition at approximately 1.6% (*w*/*v*) [7]. These properties support the potential of galactomannan as a gel-forming biopolymer, thus allowing for the development of matrices as gels [8] and films [9]. Subsequently, galactomannan was used in several applications; for example, films composed of galactomannan, glycerol, and the lectin extracted from *Cratylia mollis* seeds (CramoLL) were efficiently tested as a topical dressing for skin wounds produced in Wistar rats and demonstrated the ability to accelerate the stages of wound healing and improve the remodeling of the extracellular matrix [10]. Galactomannan was also used for the production of films containing different concentrations of the bioactive compounds lactoferrin (LF), bioactive peptides (BAPs), and phytosterols; the authors performed an extensive characterization considering the influence of immobilized biomolecules on the physicochemical properties of the films [9]. Galactomannan has also been shown to be biocompatible, nontoxic, and efficient in repairing cellular lesions [11] and showed gastroprotective activity at doses of 1.0 and 10 mg/kg, reducing the ulceration area and oxidative stress in animal models of ethanol-induced peptic ulcer [12]. More recently, a galactomannan-based gel was used to incorporate *Penicillium* sp. UCP 1286 and commercial collagenases and proved to be a viable alternative for the production of controlled-release systems [13].

Despite the above-mentioned works relating different biotechnological applications of the galactomannan from *C. grandis*, there have been no studies evaluating its toxic potential during pregnancy up to now. Reproductive studies are of great importance since fetal development is easily influenced by the prenatal environment. Thus, the intrauterine period is the critical window for the developmental plasticity of the organism [14].

Exposure to various adverse factors (e.g., improper diet, maternal diseases, and exogenous chemicals) during the intrauterine period can cause abnormalities in development and adversely affect health. Recently, some works were developed with the aim of evaluating the toxicity of these factors during pregnancy. For example, Lu et al. (2023) [14] explored the potential impacts of prenatal azithromycin exposure, while Hajar et al. (2024) [15] evaluated the developmental toxicity of the aqueous extract of *Moringa oleifera* during the prenatal period and subsequently assessed its antidepressant and anxiolytic effects using an animal model of postpartum depression induced by chronic social stress. Rodrigues et al. (2023) [16] assessed the potential embryotoxic and teratogenic effects of the aqueous extract of *Chrysobalanus icaco* leaves on maternal reproductive outcomes and fetal development in Wistar rats. Similarly, Souza et al. (2023) [17] evaluated the effect of the *Strychnos pseudoquina* aqueous extract on maternal reproductive toxicity and fetal development. The evaluation of possible maternal toxicity effects and the embryofetotoxic and teratogenic potential of polysaccharidic solutions is still under-explored; therefore, this work aimed to evaluate the toxicity of the oral administration of the galactomannan from *C. grandis* seeds during the pre-implantation and organogenesis periods in Wistar rats.

## 2. Results and Discussion

The extraction yield was 35 ± 8%, which agrees with the yield of 36 ± 8% first reported for the galactomannan from *C. grandis* seeds collected in the northeast region of Brazil [7] and more recent works with the same polysaccharide [11,12,13]. Considering this, it is then possible to mention that the methodology is reproducible and was efficiently used here to obtain the same gel-forming galactomannan already reported by our research group [7,8,9,10,11,12,13]. The FTIR spectrum confirms that the extracted material is a galactomannan (Supplementary Material).

It is worth noting that the galactomannan removal procedure employed in the present study was originally devised and characterized by Albuquerque et al. (2014) [7]. Since then, this same removal protocol has been used in various investigations by our research group involving different biological and technological applications of *Cassia grandis* galactomannan. However, although the extraction yield and FTIR profile observed in the present study align with the characteristics described for *C. grandis* galactomannan, the molar mass distribution, monosaccharide composition, polymer purity, and residual impurities were not independently determined for the experimental batch. Therefore, while the available evidence supports methodological continuity, it does not establish a complete physicochemical profile of the administered preparation. This limitation should be taken into account when interpreting the toxicological findings and extrapolating them to future biomedical applications.

### 2.1. Cytotoxicity

Biocompatibility is one of the main properties considered when evaluating the toxicity of a product; more specifically, biocompatibility can be described as the biological response displayed by an organism to a specific material. Considering that no studies have been developed to evaluate the toxic potential of the galactomannan from *C. grandis* during pregnancy up to now, the use of fibroblasts is of great importance since they are present in the earliest stages of fetal development.

**Figure 1:**
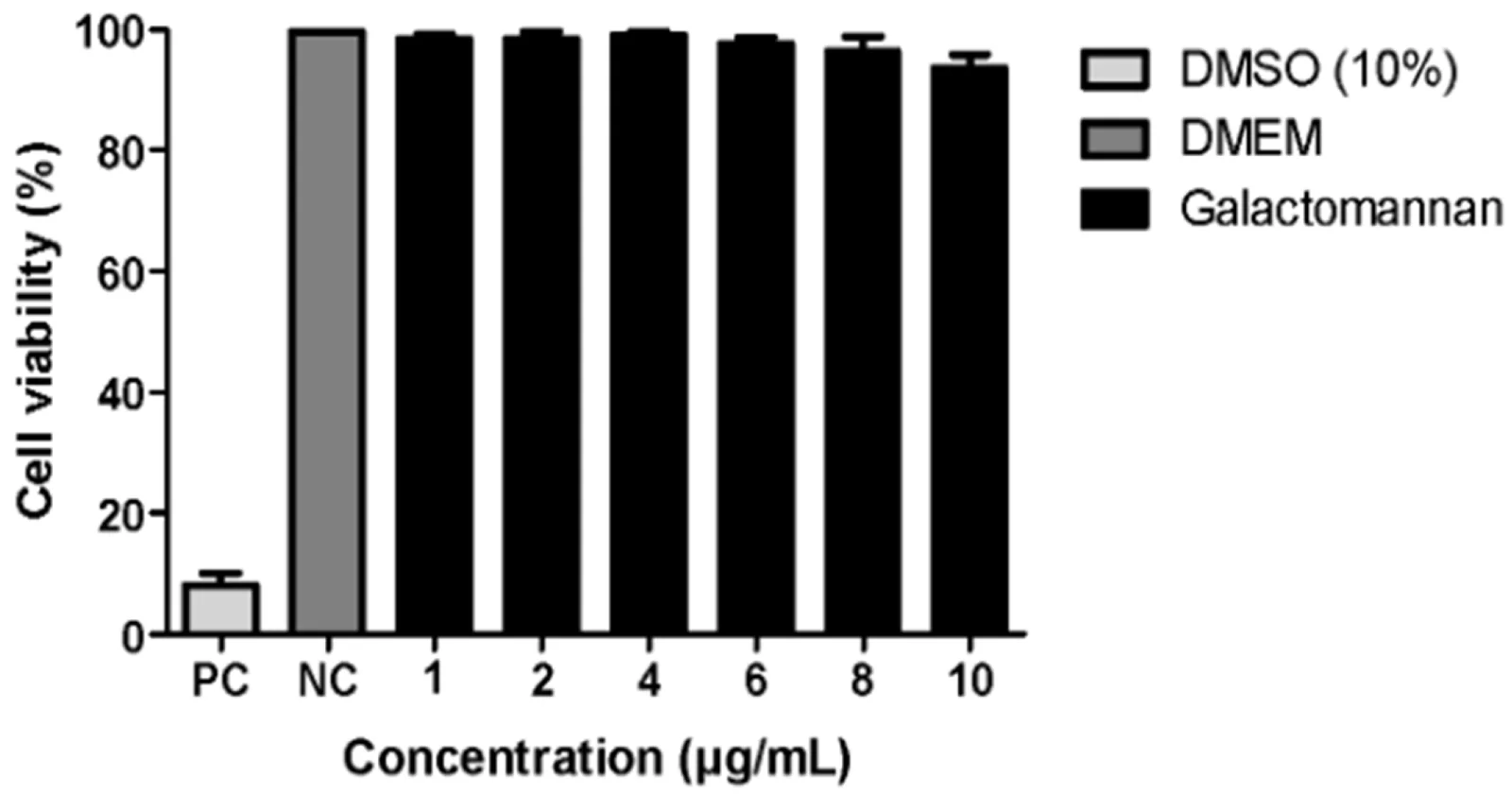
The effect of galactomannan concentrations (1–10 μg/mL) on the viability of L-929 cells, assessed by the MTT assay after 24 h of incubation at 37 °C in a 5% CO_2_ atmosphere. The positive control (PC) group received 10% DMSO, whereas the negative control (NC) group remained untreated. Data are expressed as the mean ± SD from three independent experiments (n = 3), each performed in technical triplicate. Statistical comparisons were performed using a one-way ANOVA followed by Dunnett’s multiple-comparison test against the untreated control.

In our study, galactomannan at concentrations of 1–10 µg/mL did not show cytotoxicity against L929 fibroblasts (Figure 1). Some tests were developed to evaluate the toxicity of galactomannans extracted from different sources. For example, the in vitro cytotoxicity of the galactomannans extracted from the seeds of *Dimorphandra gardneriana* Tul [18], *C. grandis* [10,11], and *Mimosa scabrella* [19] was assessed using the MTT method; all of them demonstrated no toxicity against Vero or L929 cells.

The toxicity of galactomannans was also assessed in in vivo tests, as reported in experiments with mice treated with the galactomannan extracted from the seeds of *Mimosa scabrella* [20], in rats exposed to the galactomannan from the seeds of *Adenanthera pavonina* and *Caesalpinia pulcherrina* [21], and in zebrafish (*Danio rerio*) exposed to the galactomannan from *Delonix regia* seeds [22]. However, to the best of our knowledge, the present study is the first to evaluate the toxicity of the galactomannan extracted from *C. grandis* seeds during pregnancy in rats.

### 2.2. Maternal Toxicity

The administration of galactomannan derived from *C. grandis* during the pre-implantation and organogenesis phases in Wistar rats did not induce maternal clinical signs of toxicity, including diarrhea, piloerection, stress, salivation, alterations in locomotor activity, bleeding, and mortality. Additionally, no significant differences were observed in maternal body weight gain (Table 1). Water and food consumption were monitored throughout the pre-implantation and organogenesis periods, and no statistically significant differences among groups were detected in mean daily water consumption during pre-implantation (*p* = 0.9057) or organogenesis (*p* = 0.5251). Similarly, mean daily food consumption did not differ significantly among groups during pre-implantation (*p* = 0.1239) or organogenesis (*p* = 0.6773). These comparisons were based on the individual mean consumption calculated for each pregnant dam across the respective observation period (Table 2).

Moreover, no significant alterations were detected in the weights of maternal organs, including the heart, kidneys, spleen, thymus, lungs, liver, and ovaries, during either the pre-implantation period (Table 3) or the organogenesis phase (Table 4) at any of the galactomannan doses evaluated.

The general health of mothers during the gestational period provides relevant information about the possible reproductive toxic effects of a product [23]. Maternal systemic toxicity is characterized by transient or permanent changes in mothers, including hormonal, behavioral, and homeostatic changes; these changes can be observed through the presence of clinical signs of toxicity, a reduction in body weight gain, and decreased water and feed consumption, in addition to changes in reproductive and hematological parameters and death [24,25]. Considering the treatment with the galactomannan from *C. grandis* seeds performed in this work, the oral administration of the polysaccharide at different concentrations during the pre-implantation and organogenesis periods did not induce maternal toxicity in Wistar rats.

In agreement with our results, a study developed by Deshpande et al. (2016) [26] evaluated the reproductive toxicity of the oral administration of low-molecular-weight galactomannans extracted from fenugreek seeds (*Trigonella foenum-gracum*) at doses of 250, 500 and 1000 mg/kg during pregnancy in Wistar rats. The authors did not find evidence of any mortality among treated groups during the study period. In addition, no clinical signs of maternal toxicity were observed, as well as no difference in weight gain or food consumption in treated animals when compared to the control. In another study, pregnant Sprague Dawley rats were fed daily for 10 consecutive days (7th to 16th day of pregnancy) with diets containing 10, 5 and 2.5% xylan extracted from sugarcane bagasse (*Saccharum officinarum* L.). During the study period, no mortalities were observed, nor were any clinical signs of toxicity observed. Body weights and weight gains were similar, as was food consumption, when comparing the treated and control groups [27].

### 2.3. Reproductive Performance and Histopathology of Implantation Sites and Placenta During Pre-Implantation and Organogenesis Periods

**Figure 2:**
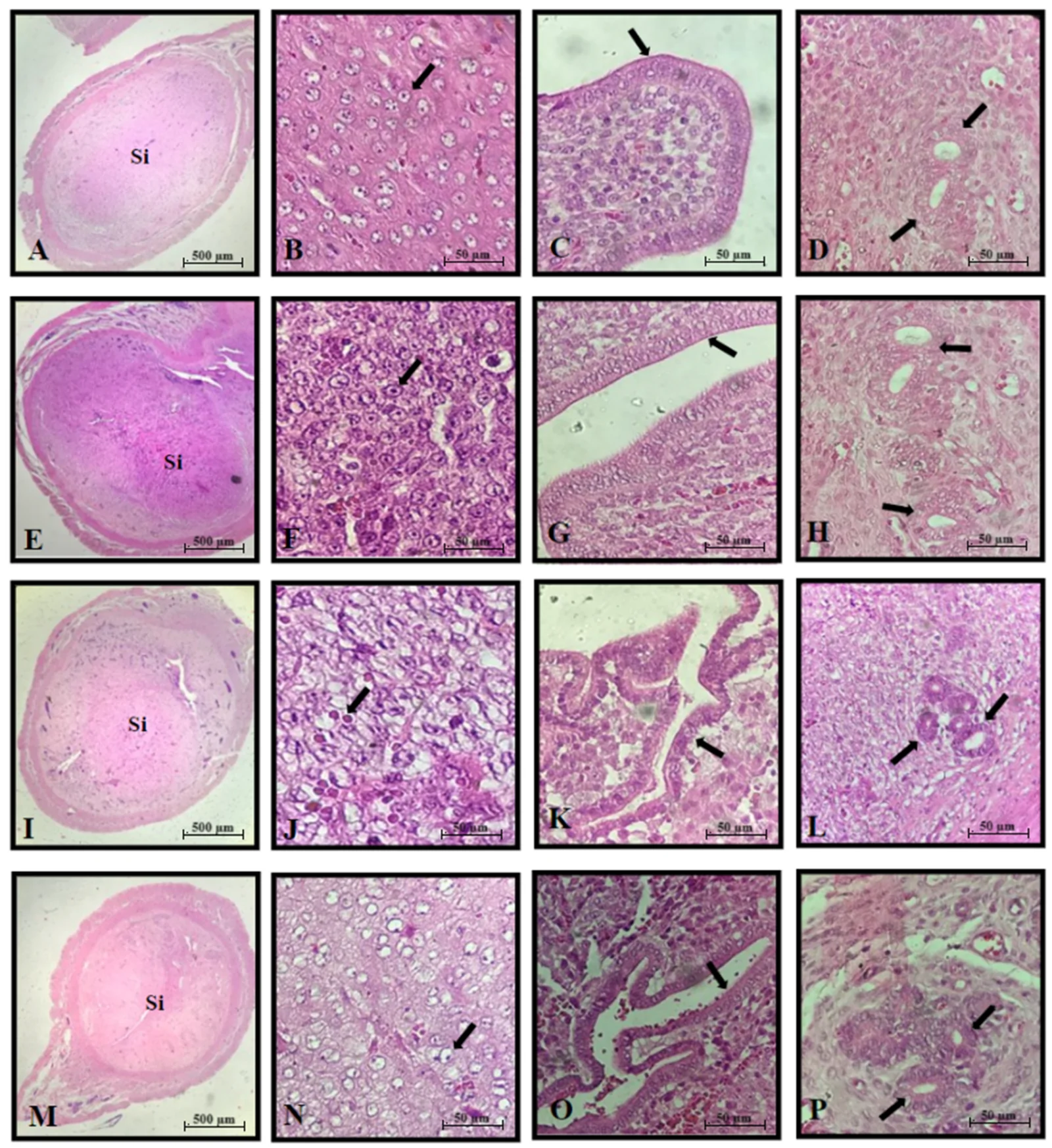
Micrograph of part of uterus of rats: (**A**–**D**) control; (**E**–**H**) 0.1 mg/kg; (**I**–**L**) 1 mg/kg; and (**M**–**P**) 10 mg/kg. (**A**,**E**,**I**,**M**): Implantation site (Si) completely embedded in uterine wall. HE ± 40×. (**B**,**F**,**J**,**N**): Trophoblasts (arrow). HE ± 400×. (**C**,**G**,**K**,**O**): Luminal epithelium (arrow). HE ± 400×. (**D**,**H**,**L**,**P**): Endometrial glands (arrows). HE ± 400×.

With respect to the pre-implantation phase, no statistically significant differences were detected between treated and control groups regarding the number of corpora lutea, the number of implantation sites, the implantation index or the rate of pre-implantation loss (Table 5). The histopathological evaluation of the implantation sites (ISs) revealed that, in both treated and control animals, the ISs were fully embedded within the uterine wall. These structures were composed predominantly of trophoblastic cells, some exhibiting mitotic figures, as well as polyploid cytotrophoblasts and marked vascularization. The luminal epithelium displayed a simple columnar pattern, and numerous endometrial glands were observed within the decidual tissue (Figure 2).

When the litter was considered the experimental unit, no statistically significant differences among groups were detected for the number of live fetuses, numbers of male and female fetuses, sex ratio, implantation sites, resorption sites, corpora lutea, implantation index, or post-implantation loss rate (Table 6). A significant overall group effect was observed for litter-mean fetal body weight (one-way ANOVA, *p* = 0.0168). However, no galactomannan-treated group differed significantly from the control group in the post hoc analysis. The only significant pairwise difference was observed between the 0.1 and 10 mg/kg groups (Tukey’s test, *p* = 0.0162).

The biological significance of this finding in developmental toxicity studies remains uncertain, as fetal weight can be influenced by various factors, including litter size, fetal sex, and intrauterine variability [28]. Furthermore, the limited number of litters per group (n = 5) reduces the precision of effect estimates and the ability to detect subtle developmental effects. Therefore, although the observed pattern does not provide clear evidence of the treatment-related impairment of fetal growth, a potential developmental effect cannot be definitively ruled out.

**Figure 3:**
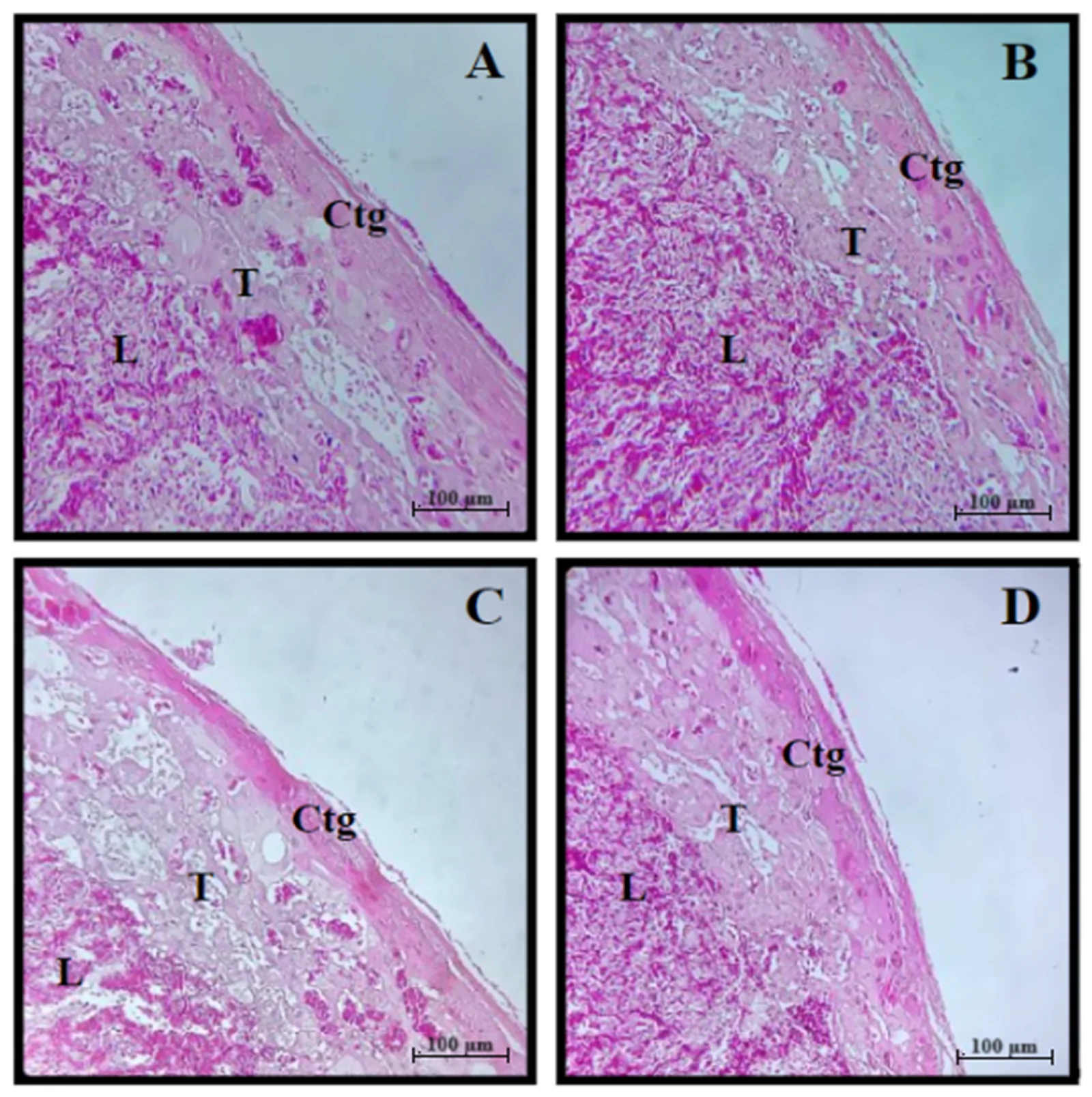
Micrograph of placentas. (**A**) control; (**B**) 0.1 mg/Kg of galactomannan; (**C**) 1 mg/Kg of galactomannan; and (**D**) 10 mg/Kg of galactomannan. L: labyrinth; T: trophospongium; Ctg = trophoblastic giant cells. HE stain.

Concerning placental histopathology, both treated and control groups exhibited a well-developed placental disk organized into the labyrinth zone, trophospongium, and a layer of giant trophoblastic cells. In the labyrinth layer, which represents the outermost and thickest region, numerous lacunar spaces containing maternal and fetal blood vessels were identified. The trophospongium layer consisted predominantly of undifferentiated trophoblastic cells. In the innermost region, giant trophoblastic cells were observed to be intermingled with the decidual tissue (Figure 3).

The evaluation of female reproductive performance is an important parameter for analyzing the reproductive toxicity of a product [16,29]. Embryonic implantation is the process by which the blastocyst reaches a stable position in the endometrium, thus allowing for the maintenance of an efficient system of metabolic exchanges between the endometrium and the embryo [30]. In rats, this process occurs from the 1st to the 5th day of pregnancy [31], a period in which the blastocyst is sensitive to chemical exposure, which can lead to its death [32].

The implantation index is considered an indicator of the correct implantation of the blastocyst in the uterus [33], while the resorption rate and pre-implantation loss establish a correlation between the number of blastocysts implanted and non-developed ones [34]. In this study, it was observed that the administration of galactomannan at different concentrations did not change the rates of pre-implantation, implantation, and resorption losses. These results corroborate the results obtained in the histopathological analysis, which demonstrated well-preserved ISs, fully inserted into the uterine wall and well developed. Based on these findings, it is possible to suggest that the administration of galactomannan does not interfere with reproductive parameters during the pre-implantation period.

During gestational development, chemical, physical, and environmental factors may cause embryofetal toxicity or pre- and post-implantation losses, developmental delay, malformations, and even fetal death [14,35]. Our results showed that the oral administration of galactomannan in rats during the organogenesis period did not induce fetal toxicity since it did not change the number of live and dead fetuses, number of implantations, rate of post-implantation losses, or number of previous and late resorptions.

The absence of embryofetotoxicity can also be evaluated by maintaining the sex ratio of the offspring since exposure to toxic products during the organogenesis period can delay the animals’ development and sexual differentiation [36]. Thus, the similarity observed in the sex ratio of the offspring of rats treated with galactomannan at different concentrations and those of the control group (treated with water) suggests the absence of embryofetotoxic effects on the litters.

Finally, an adequate nutrient supply through the placenta can be directly linked to fetal development in the uterus, while a decrease in placental size is closely related to the incidence of fetal distress [37].

Considering that no significant differences were observed in placental weight or the placental index among the groups, and that the histopathological evaluation did not reveal apparent treatment-related morphological alterations, these findings do not suggest an adverse effect of galactomannan on the placental parameters evaluated under the experimental conditions of this study [38].

In a comparative study, the evaluation of embryofetotoxic changes in the polysaccharide xylan extracted from sugarcane bagasse (*Saccharum officinarum* L.) in pregnant Sprague Dawley rats did not show the occurrence of abortion or premature birth. The weights of the pregnant uterus and placentas, as well as the average number of implantations, did not show differences between the treated and control groups. Furthermore, the same study showed that xylan administered by gavage for five consecutive days at concentrations of 1.25, 2.50 and 5.00 g/kg in male Kunming mice was not able to alter the morphology of the mice’s sperm, demonstrating its low toxic potential in relation to the reproductive parameters of females and males [27].

Another developmental toxicity study in pregnant rats that received 250, 500 or 1000 mg of cassia gum/kg of body weight per day via gavage during the period of organogenesis did not show any impact on the number of resorptions or dead embryos or on the weight and length of the fetuses [39]. Similarly, rats treated with galactomannan extracted from *Trigonella foenum-gracum* at doses of 250, 500 and 1000 mg/kg did not show significant differences between the number of live fetuses, the quantification of corpora lutea and implantation sites, in addition to not having shown any changes in the rates of resorption and pre- and post-implantation losses between the treated groups when compared to the control [26].

A study of the embryofetal toxicity potential of the polysaccharide extracted from *Cyamopsis tetragonoloba* seeds in female Osborne–Mendel rats fed with gum in the diet at concentrations of 0, 1, 3, 4, 7.5 or 15% in proportions of 0, 0.7, 1.4, 2.7, 5.2 or 11.8 g/kg of body weight/day, respectively, during pregnancy did not show any changes in the number of corpora lutea and implantations in the treated groups, when compared to the control. However, a slight reduction in the number of viable fetuses was observed in the group treated with 15% but without a statistical difference. It was concluded that the addition of *C. tetragonoloba* (guar) gum to the diet had no embryofetal toxicity effects [40].

Borzelleca and collaborators (1993) [41] investigated the multigenerational impact of the administration of *Caesalpinia spinosa* gum to Charles River CD albino rats at a dietary level of 5% (50,000 ppm) for three successive generations. During the entire experiment, no consistent and statistically related adverse effects were observed in relation to the toxicity of the substance, including mortality, food consumption, body weight gain, general health and behavior. Furthermore, no changes in reproductive performance and mating indices were observed between the control and gum-treated groups. The macroscopic examination of the parental animals and lateral examination and microscopic examination of the progeny tissues did not show any abnormalities. It is concluded that *C. spinosa* gum was not capable of causing an adverse effect on the reproductive performance or development of the progeny.

### 2.4. Evaluation of External and Internal Fetal Malformations (Skeletal and Visceral)

**Figure 4:**
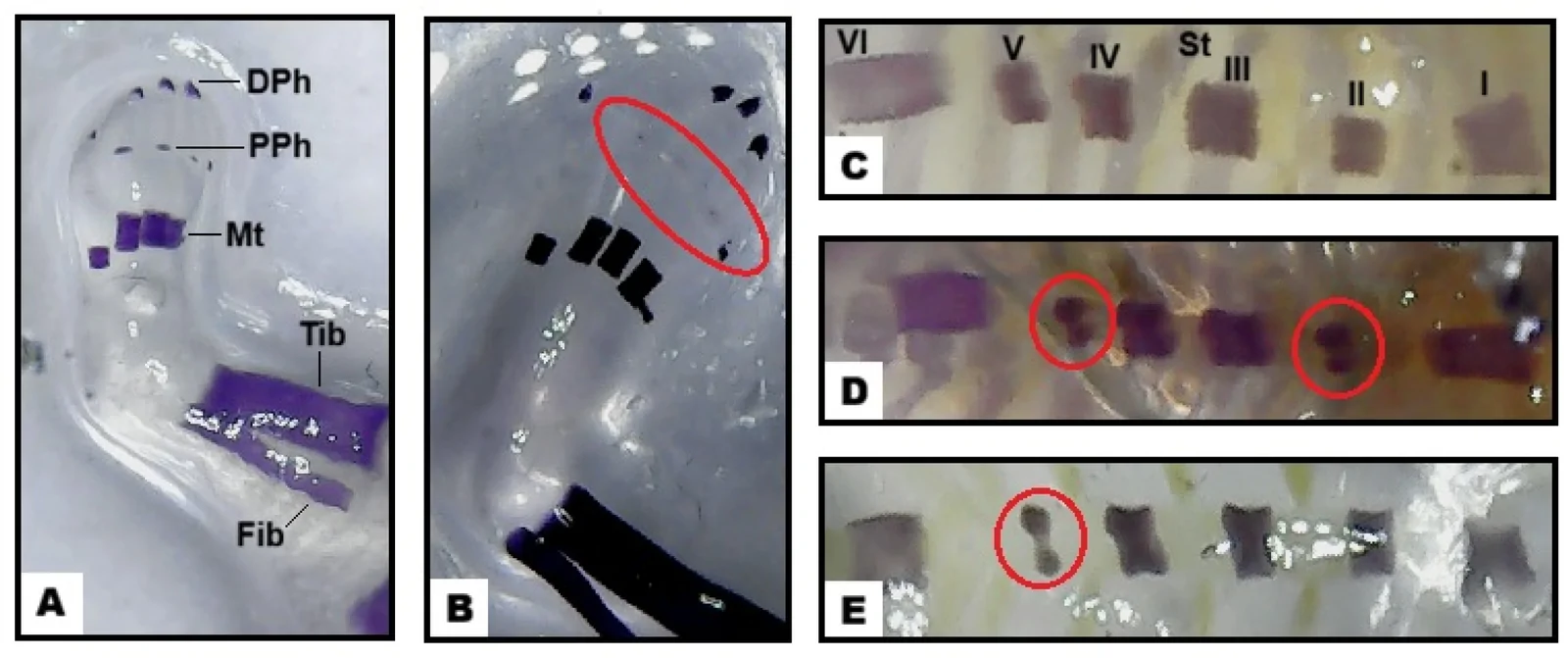
Representative skeletal findings of the normal and incomplete ossification of the proximal phalanges of the hindlimbs and sternebrae in fetuses from control and galactomannan-treated groups. (**A**) The normal ossification of the hindlimb skeletal structures in a fetus from the control group. (**B**) The incomplete ossification of the proximal phalanges of the hindlimb in a fetus from the 1 mg/kg group, highlighted by a red oval. (**C**) The normal ossification of the sternebrae in a fetus from the 0.1 mg/kg group. (**D**) The incomplete ossification of the second (II) and fifth (V) sternebrae in a fetus from the 10 mg/kg group, indicated by red circles. (**E**) The incomplete ossification of the fifth (V) sternebra in a fetus from the control group, indicated by a red circle. Roman numerals I–VI indicate the first through sixth sternebrae, respectively. Abbreviations: DPh, distal phalanges; PPh, proximal phalanges; Mt, metatarsals; Tib, tibia; Fib, fibula; St, sternebrae.

No external malformations were observed in the fetuses from either the control or galactomannan-treated groups. Similarly, visceral examination did not reveal apparent structural abnormalities in any experimental group. Skeletal examination identified the incomplete ossification of the proximal phalanges and sternebrae (Figure 4) at low frequencies in both control and treated groups (Table 7). When the litter was considered the experimental unit, no statistically significant differences among groups were detected in the litter-level proportions of fetuses presenting the incomplete ossification of the proximal phalanges (Kruskal–Wallis, *p* = 0.9024) or sternebrae (*p* = 0.7666). No other skeletal findings were observed in any group.

The effects of teratogenic agents usually manifest during the organogenesis period since the structures of the embryos undergoing development and differentiation are more susceptible to the effects of teratogens [42]. In our study, no external malformations were observed in the litter, nor were there any visceral structural changes between the fetuses of all of the groups. The incomplete ossification of the proximal phalanges of the lower limbs and sternebrae was observed at low frequencies in both control and galactomannan-treated groups, with no statistically significant differences among groups when the litter was considered the experimental unit. At this moment, it is important to say that the incomplete ossification of the sternebrium and proximal phalanges of the lower limbs is characterized as a variation and not a malformation.

According to the terminologies and concepts adopted in developmental toxicology, variations are generally reversible changes after birth, while malformations are permanent changes that can harm the health and survival of a specimen [43,44]. According to Fritz and Hess (1970) [45], the presence of variations in the sternum bones in rodents is relatively common, and approximately 95% to 100% of the sternebrae are ossified after birth. According to Carney and Kimmel (2007) [46], incomplete or delayed ossification is a common finding in developmental toxicity studies, particularly in skeletal structures that undergo rapid ossification during late gestation, including the phalanges and sternebrae. Such findings may represent transient variations in skeletal maturation rather than permanent structural abnormalities and should be interpreted in the context of their incidence, distribution, and associated maternal and fetal findings. In the present study, incomplete ossification was predominant in the fifth sternebra.

In relation to the appendicular skeleton, delays in the ossification of the proximal phalanges of the lower limbs were observed. These findings are also considered variations and refer to a decrease in the amount of mineralized bone in relation to that expected for a given age. This result is common in developmental toxicology studies, mainly because fetuses are evaluated on the 21st day of gestation, when the ossification process is not yet complete for specific regions [47,48,49,50].

The selection of the doses evaluated in the present study (0.1, 1.0, and 10 mg/kg) was supported by previous experimental evidence demonstrating the biological activity of plant-derived polysaccharides at relatively low doses. Notably, the oral administration of galactomannan extracted from *Cassia grandis* seeds at 1.0 and 10 mg/kg has previously demonstrated significant gastroprotective activity in Wistar rats, reducing gastric mucosal injury and oxidative stress [12]. Similarly, Marques et al. (2019) [51] reported that galactomannan isolated from *Caesalpinia pulcherrima* seeds exhibited gastroprotective activity against indomethacin-induced gastric injury in mice at doses of 3, 10, and 30 mg/kg, with protective effects observed at 10 mg/kg. Furthermore, Dallazen et al. (2024) [52] demonstrated the antinociceptive and anti-inflammatory activities of an arabinan-rich pectic polysaccharide fraction from *Malpighia emarginata* at relatively low doses, including those investigated in the present study. Collectively, these findings provide a pharmacological rationale for investigating the selected dose range, particularly because it encompasses exposure levels previously associated with the biological effects of the same galactomannan. Nevertheless, the selected doses are substantially lower than those commonly employed in comprehensive reproductive toxicity studies and were not established based on anticipated human exposure levels. Therefore, although the present findings contribute preliminary information regarding maternal and developmental responses within a biologically relevant experimental dose range, they cannot be extrapolated to higher exposures or to future biomedical applications without additional investigations. Studies employing broader dose ranges, exposure scenarios relevant to specific formulations, and appropriate reproductive toxicity protocols are necessary to establish a more comprehensive safety profile.

A further consideration concerns the physical state of the galactomannan preparation administered in this study. Although *C. grandis* galactomannan has been investigated for the development of gel-based biomedical materials, the present reproductive toxicity assessment was conducted using aqueous solutions of the isolated polysaccharide rather than pre-formed gels. This approach aimed to investigate the biological response to the galactomannan itself, without the influence of additional formulation components. Incorporation into gel-based systems is known to modify the polysaccharide’s physicochemical behavior—including aspects such as swelling, gastrointestinal transit, interactions with biological components, and the release or availability of intrinsic constituents. Consequently, the results obtained with galactomannan in an aqueous medium cannot be directly extrapolated to gels or other biomedical formulations. Further investigations using the intended formulations and clinically relevant routes of administration are warranted to evaluate their specific safety profiles. Furthermore, an important limitation of the present study is the relatively small number of pregnant females per group (n = 5), which limits the statistical power to detect small or moderate treatment-related effects. Therefore, the absence of statistically significant differences should not be interpreted as definitive evidence of the absence of maternal or developmental toxicity, and the present findings should be considered exploratory.

## 3. Conclusions

Under the experimental conditions evaluated, the oral administration of *Cassia grandis* galactomannan at doses of 0.1, 1.0, and 10 mg/kg did not result in detectable treatment-related adverse effects on the maternal and developmental parameters assessed during the pre-implantation and organogenesis periods in Wistar rats. Although a statistically significant difference in fetal body weight was observed between the lowest- and highest-dose groups, neither group differed significantly from the control, and no consistent dose-dependent pattern of fetal growth impairment was identified. No treatment-related increase in the fetal skeletal findings evaluated was observed. However, considering the exploratory nature of this study and the limited number of pregnant dams per group, these findings should not be interpreted as definitive evidence of the absence of maternal or developmental toxicity. Further studies with adequately powered experimental designs are required to confirm the reproductive and developmental safety profile of this polysaccharide. Furthermore, since this study evaluated aqueous galactomannan solutions rather than pre-formed gels, the results cannot be directly extrapolated to gel-based biomedical formulations. Despite our efforts to report, for the first time, on the toxicity effects of the galactomannan from *C. grandis*, we know that some considerable challenging issues have yet to be resolved, for example, its toxicity in different experimental models and/or at different concentrations. As a goal in our research group, we plan to perform other experiments and evaluate the safety of galactomannan’s administration.

## 4. Materials and Methods

### 4.1. Extraction and Solubilization of Galactomannan from C. grandis

*C. grandis* seeds were harvested in the state of Pernambuco, Brazil (16°19′36″ S; 48°57′10″ W), as conducted in previous studies by our research group [11,12,13]. In summary, the extraction protocol started with the immersion of dried seeds in distilled water at a 1:5 (*w*/*v*) ratio followed by heating to 100 °C for 1 h, maintaining a temperature of 25 °C for an additional 18 h to aid in husk removal. Subsequently, the endosperm and germ were homogenized in a blender with 0.1 M NaCl at 5% (*w*/*v*) at 25 °C. The mixture was filtered through a screen-printing mesh and subjected to precipitation with cold 46% ethanol at a 1:3 (*v*/*v*) ratio for 24 h. The resulting precipitate was rinsed with absolute ethanol (100%) at 1:3 (*v*/*v*) and then washed twice with analytical-grade acetone (PA) at the same proportion, with filtration through a screen-printing cloth performed between each washing step. The galactomannan precipitate was subsequently dried in an oven at 100 °C, ground into a fine powder, and stored in a dry environment until further use. The yield of the extraction was obtained by dividing the final mass of galactomannan (Mf) by the initial mass of dry seeds (Mi) and expressed as % *w*/*w*; this process was carried out in triplicate and the results reported as the mean ± standard deviation.

For the in vivo experiments, a 1.0% (*w*/*v*; 10 mg/mL) aqueous galactomannan preparation was obtained using autoclaved distilled water under continuous stirring at 500 rpm for 18 h at 25 ± 2 °C. It is important to mention that the galactomannan used in this study is a gel-forming polysaccharide with previously reported concentration-dependent rheological behavior and gelation potential [7]. Thus, we aimed to obtain a preparation sufficiently homogeneous and fluid for reproducible oral gavage; in this case, a homogeneous aqueous galactomannan preparation was preferred rather than a pre-formed gel.

The 1.0% (*w*/*v*; 10 mg/mL) concentration was prepared, stored at 2–8 °C and replaced every three days, and used for the highest-dose group; then, it was serially diluted with distilled water to obtain concentrations of 1.0 and 0.1 mg/mL. All preparations were administered once daily by oral gavage at a constant volume of 1 mL/kg body weight (0.1 mL/100 g), adjusted according to the individual body weight of each animal. Accordingly, concentrations of 0.1, 1.0, and 10 mg/mL corresponded to doses of 0.1, 1.0, and 10 mg/kg body weight, respectively. Control animals received distilled water at the same administration volume.

The use of a low and constant administration volume was intended to minimize potential physiological interference associated with repeated gavage during pregnancy while maintaining identical administration volumes across experimental groups. The selected doses provided three logarithmically spaced exposure levels (0.1, 1.0, and 10 mg/kg) for the exploratory assessment of maternal and developmental effects under the experimental conditions employed. This study was not designed to establish a maximum tolerated dose or a No-Observed-Adverse-Effect Level (NOAEL).

### 4.2. Cell Culture

The fibroblast cell line (L929) was maintained in Dulbecco’s Modified Eagle Medium (DMEM) supplemented with 10% fetal bovine serum, 1% glutamine, and antibiotics (100 U/mL penicillin and 10 µg/mL streptomycin), at 37 °C in a humidified atmosphere containing 5% CO_2_. Cell manipulation was performed under aseptic conditions in a vertical laminar flow hood, and cultures were grown in 25 cm^2^ plastic culture flasks with a total capacity of 50 mL.

### 4.3. Effect of Galactomannan on Cell Viability

The cytotoxic potential of galactomannan was assessed using the MTT assay, as described by Mosmann (1983) [53]. Fibroblasts (7 × 10^3^ cells/well) were seeded into 96-well plates and exposed to galactomannan at concentrations ranging from 1 to 10 µg/mL. Following a 24 h incubation at 37 °C in a CO_2_ incubator, MTT solution (5 mg/mL; [3-(4,5-dimethylthiazol-2-yl)-2,5-diphenyl tetrazolium bromide]) was added to each well, and the plates were further incubated for 4 h at 37 °C under 5% CO_2_. After this period, the supernatant was carefully removed, and 100 µL of dimethyl sulfoxide (DMSO) was added to dissolve the formazan crystals formed. The absorbance of each well was measured at 540 nm. Cell viability and proliferation rates were calculated relative to the control group using the following formula: (absorbance of treated cells/absorbance of untreated cells) × 100%. The assay was performed in three independent experiments, with each galactomannan concentration and control condition tested in technical triplicate in each experiment.

### 4.4. Animals

Albino Wistar rats (*Rattus norvegicus*), weighing 180–240 g, obtained from the vivarium of the Federal Rural University of Pernambuco, were housed under controlled environmental conditions, including a temperature of 22 ± 2 °C, a 12 h light–dark cycle, and a relative humidity of 60 ± 1%, with free access to water and food. All experimental procedures were conducted in accordance with ethical guidelines and were approved by the Animal Use Ethics Committee of the Federal Rural University of Pernambuco (Protocol number: 2589020720).

### 4.5. Experimental Protocol

The mating procedure and determination of the onset of pregnancy were based on procedures described in OECD Test Guideline 421 (OECD, 2025) [36], with adaptations according to the experimental design of the present study. Nulliparous females were paired with adult males at a 2:1 ratio at the onset of the dark phase. After 12 h (corresponding to the beginning of the light phase), vaginal lavage was performed using 0.9% NaCl, and the samples were examined under light microscopy. Mating was confirmed by the detection of spermatozoa in the lavage fluid and by determining the estrus stage of the estrous cycle. The identification of sperm was designated as gestational day 0 [54]. Breeding procedures continued until the required number of pregnant females was reached (n = 40).

Following the confirmation of mating, the animals were randomly allocated into eight experimental groups (n = 5/group). Four groups received treatment during the pre-implantation period (gestational days 1–6), while the remaining four groups were treated during the organogenesis phase (gestational days 6–15). The control animals (group 1) received distilled water (1 mL/kg body weight), while the treatment groups received galactomannan at doses of 0.1, 1.0, or 10 mg/kg body weight (groups 2, 3, and 4, respectively). All treatments were administered once daily by oral gavage at a constant volume of 1 mL/kg, adjusted according to the individual body weight of each animal.

Pregnant rats were randomly allocated to the experimental groups by the simple drawing of lots, with five dams per group. No animals or litters were excluded after group allocation. The number of animals was selected considering the exploratory nature of this study and the principle of Reduction within the 3Rs (Replacement, Reduction, and Refinement), aiming to minimize animal use. No formal a priori sample-size calculation was performed.

### 4.6. Analysis of Maternal Toxicity

The animals were monitored daily for the assessment of clinical signs indicative of toxicity, including diarrhea, piloerection, behavioral stress, alterations in motor activity, and bleeding. In addition, food and water intake, as well as body weight progression, were recorded throughout the experimental period [36].

### 4.7. Evaluation of Maternal Reproductive Performance

#### 4.7.1. Pre-Implantation Period

On gestational day 7, the rats were anesthetized via the intraperitoneal (i.p.) administration of ketamine hydrochloride (60 mg/kg) combined with xylazine hydrochloride (6 mg/kg). Subsequently, a laparotomy was carried out, and euthanasia was completed by cardiac incision. The uterine horns were excised for the determination of the number of implantation sites. The ovaries were also removed and weighed, and the corpora lutea were quantified. The percentage of pre-implantation loss was calculated according to the following equation: Pre-implantation loss (%) = [(number of corpora lutea − number of implantations)/number of corpora lutea] × 100. Maternal organs, including the thymus, heart, lungs, liver, kidneys, and spleen, were harvested, weighed, and examined for gross morphological alterations. Relative organ weight was determined using the following formula: (organ weight/maternal body weight on the day of euthanasia) × 100.

#### 4.7.2. Organogenesis Period

On gestational day 21, the rats were anesthetized following the protocol previously described in subtopic 4.7.1. A laparotomy was subsequently performed, and euthanasia was completed by cardiac incision. The uterine horns were examined macroscopically to identify the presence of live or dead fetuses, as well as early or late resorptions. The following reproductive parameters were determined: implantation index [(total number of implantation sites/total number of corpora lutea) × 100], resorption index [(total number of resorption sites/total number of implantation sites) × 100], and post-implantation loss rate {[(number of implantation sites − number of live fetuses)/number of implantation sites] × 100}. After hysterectomy, the fetuses were weighed and sexed. The placental index (placental weight/fetal weight) and sex ratio (number of males/number of females) were also calculated. Maternal organs, including the thymus, heart, lungs, liver, kidneys, and spleen, were excised, weighed, and assessed for gross morphological abnormalities.

### 4.8. Analysis of Malformations

The fetuses underwent external examination in accordance with the criteria established by Wilson (1965) [55], including a detailed assessment of the eyes, oral cavity, ear positioning, cranial morphology, forelimbs and hindlimbs, anal opening, and tail. Following the external evaluation, half of each litter was fixed in Bodian solution for subsequent visceral analysis using the serial sectioning technique, which involves a thorough examination of the transverse sections of the head, thorax, and pelvic regions, as described by Wilson (1965) [55]. The remaining half of each litter was processed according to methods adapted from Manson et al. (1982) [56], cleared, and stained with 1% alizarin red solution following the protocol of Staples and Schnell (1964) [57]. The nomenclature adopted for skeletal abnormalities was based on the recommendations of the International Federation of Teratology Societies (Makris et al., 2009) [58]. Both skeletal and visceral evaluations were conducted using a Leica^®^ EZ4 W stereomicroscope (Leica, Wetzlar, Germany). Fetal, visceral, and skeletal examinations were performed by investigators blinded to the treatment-group allocation.

### 4.9. Histological Analysis of Implantation Sites

Following hysterectomy, implantation sites and placental tissues were immersed in 10% (*v*/*v*) buffered formalin and fixed for 24 h. After fixation, the specimens were processed according to standard histopathological procedures, and 5 µm thick sections were prepared. The histological sections were examined after hematoxylin and eosin (H&E) staining. Microscopic evaluation was carried out using an Olympus^®^ BX50 microscope (Olympus Corporation, Tokyo, Japan) coupled to a Sony^®^ video system (Sony, Tokyo, Japan). Histological evaluation was performed in all five dams from each experimental group. Three placentas and three implantation sites per dam were examined, with three histological sections evaluated per placenta and per implantation site, resulting in nine placental sections and nine implantation-site sections per dam.

### 4.10. Statistical Analysis

Statistical analyses were performed using Prism 6.0^®^ software. Data are presented as the mean ± standard deviation (SD) or as the median, as appropriate. Data distribution was assessed using the Shapiro–Wilk test, and homogeneity of variances was evaluated using Bartlett’s test. For continuous variables meeting the assumptions for parametric analysis, comparisons among groups were performed using a one-way analysis of variance (ANOVA), followed by Tukey’s multiple-comparison test when comparisons among all groups were required. For the in vitro cytotoxicity assay, a one-way ANOVA followed by Dunnett’s multiple-comparison test was used to compare each galactomannan concentration with the untreated control. When the assumptions for parametric analysis were not met, the Kruskal–Wallis test followed by Dunn’s multiple-comparison test was used. Statistical significance was defined as *p* < 0.05.

For reproductive and developmental outcomes, a pregnant dam/litter was considered the experimental unit (n = 5 litters per group), and individual fetuses within the same litter were not considered independent observations. For fetal body weight, the mean fetal weight was first calculated separately for each litter, and litter means were subsequently used for group-level statistical comparisons. The sex ratio and reproductive indices were likewise calculated at the litter level. For the skeletal findings, fetal-level incidences were reported descriptively. For statistical comparisons, the proportion of affected fetuses was calculated separately for each litter, and these litter-level proportions were compared among groups using the Kruskal–Wallis test. Findings with zero incidence in all experimental groups were not subjected to statistical testing.

## Figures and Tables

**Table 1:** Maternal body weight during galactomannan administration throughout the pre-implantation period and the organogenesis phase.

	Pre-Implantation Weight (g)	
	Day 0	Day 6
Control	203.75 ± 8.32 ^a^	215.50 ± 9.32 ^a^
Galactomannan 0.1 mg/kg	200.25 ± 9.05 ^a^	218.00 ± 11.13 ^a^
Galactomannan 1.0 mg/kg	196.60 ± 9.71 ^a^	216.40 ± 10.80 ^a^
Galactomannan 10 mg/Kg	199.00 ± 7.34 ^a^	221.25 ± 9.70 ^a^
	Organogenesis Weight (g)	
	Day 6	Day 21
Control	215.25 ± 10.68 ^a^	308.5 ± 10.96 ^a^
Galactomannan 0.1 mg/kg	207.50 ± 9.60 ^a^	310.5 ± 10.70 ^a^
Galactomannan 1.0 mg/kg	219.00 ± 9.88 ^a^	314.75 ± 11.02 ^a^
Galactomannan 10 mg/kg	212.50 ± 10.97 ^a^	305.60 ± 9.00 ^a^

In the pre-implantation study, treatment was administered from gestational days 1 to 6, and body weight was recorded from days 0 to 6. In the organogenesis study, treatment was administered from gestational days 6 to 15, whereas maternal body weight was monitored from days 6 to 21. Means followed by the same letter within each column are not significantly different according to a one-way ANOVA followed by Tukey’s post hoc test. Data are expressed as the mean ± standard deviation (n = 5 per group).

**Table 2:** The water and food consumption of pregnant Wistar rats treated with galactomannan during the pre-implantation and organogenesis periods.

Parameter	GD	Control	0.1 mg/kg	1 mg/kg	10 mg/kg
	**Pre-implantation period**				
**Water consumption (mL/day)**	1	24.80 ± 5.18	25.30 ± 5.67	26.50 ± 3.08	27.46 ± 9.44
	3	32.60 ± 5.59	30.70 ± 8.39	31.64 ± 9.02	33.48 ± 6.99
	7	38.70 ± 3.90	39.20 ± 5.76	37.70 ± 4.60	39.96 ± 7.46
**Food consumption (g/day)**	1	16.50 ± 2.24	18.03 ± 1.85	17.72 ± 2.98	20.00 ± 6.52
	3	22.48 ± 2.91	23.88 ± 5.33	19.82 ± 1.30	23.50 ± 3.67
	7	27.60 ± 2.88	26.33 ± 5.12	25.30 ± 3.37	28.70 ± 6.99
	**Organogenesis period**				
**Water consumption (mL/day)**	6	42.20 ± 3.56	40.20 ± 3.35	41.60 ± 2.70	41.00 ± 2.83
	14	49.80 ± 12.30	48.72 ± 10.44	45.60 ± 7.02	50.60 ± 8.95
	21	56.40 ± 5.86	64.60 ± 4.22	60.80 ± 7.69	56.98 ± 5.04
**Food consumption (g/day)**	6	19.60 ± 1.14	20.40 ± 1.14	19.40 ± 1.14	19.60 ± 1.52
	14	20.40 ± 1.75	18.90 ± 4.53	23.08 ± 3.28	25.64 ± 4.98
	21	26.20 ± 1.30	27.00 ± 3.00	32.40 ± 5.55	30.00 ± 6.04

Data are expressed as the mean ± standard deviation (SD) of five pregnant rats per group (n = 5). Water and food consumption are expressed in mL/animal/day and g/animal/day, respectively. GD: gestational day. Galactomannan was administered orally at doses of 0.1, 1, and 10 mg/kg during GD1–GD6 (pre-implantation) or GD6–GD15 (organogenesis). A pregnant dam was considered the experimental unit. No statistically significant differences among groups were detected in mean daily water or food consumption over the respective observation periods (one-way ANOVA, *p* > 0.05).

**Table 3:** The effect of oral galactomannan administration on the absolute (g) and relative (%) organ weights of Wistar rats during the pre-implantation period (gestational days 1–6).

Organs	Control	Galactomannan		
		0.1 mg/kg	1 mg/kg	10 mg/kg
Liver (g)	8.97 ± 0.72 ^a^	8.81 ± 0.64 ^a^	9.07 ± 0.93 ^a^	8.80 ± 0.75 ^a^
Liver (%)	4.69 ± 0.43 ^a^	4.53 ± 0.46 ^a^	4.63 ± 0.53 ^a^	4.54 ± 0.41 ^a^
Kidney (g)	1.57 ± 0.15 ^a^	1.62 ± 0.09 ^a^	1.65 ± 0.03 ^a^	1.55 ± 0.19 ^a^
Kidney (%)	0.73 ± 0.09 ^a^	0.83 ± 0.07 ^a^	0.81 ± 0.04 ^a^	0.79 ± 0.03 ^a^
Spleen (g)	0.82 ± 0.11 ^a^	0.86 ± 0.08 ^a^	0.87 ± 0.15 ^a^	0.95 ± 0.20 ^a^
Spleen (%)	0.38 ± 0.05 ^a^	0.44 ± 0.05 ^a^	0.42 ± 0.06 ^a^	0.48 ± 0.08 ^a^
Heart (g)	0.78 ± 0.08 ^a^	0.73 ± 0.07 ^a^	0.73 ± 0.06 ^a^	0.69 ± 0.06 ^a^
Heart (%)	0.36 ± 0.05 ^a^	0.37 ± 0.04 ^a^	0.36 ± 0.01 ^a^	0.35 ± 0.02 ^a^
Lungs (g)	1.17 ± 0.17 ^a^	1.33 ± 0.16 ^a^	1.24 ± 0.15 ^a^	1.22 ± 0.08 ^a^
Lungs (%)	0.54 ± 0.10 ^a^	0.68 ± 0.11 ^a^	0.61 ± 0.05 ^a^	0.63 ± 0.07 ^a^
Thymus (g)	0.40 ± 0.09 ^a^	0.56 ± 0.09 ^a^	0.47 ± 0.16 ^a^	0.42 ± 0.09 ^a^
Thymus (%)	0.18 ± 0.04 ^a^	0.22 ± 0.04 ^a^	0.23 ± 0.06 ^a^	0.21 ± 0.02 ^a^
Ovary (g)	0.11 ± 0.00 ^a^	0.12 ± 0.02 ^a^	0.12 ± 0.02 ^a^	0.10 ± 0.01 ^a^
Ovary (%)	0.04 ± 0.00 ^a^	0.06 ± 0.01 ^a^	0.06 ± 0.01 ^a^	0.05 ± 0.00 ^a^
Uterus (g)	0.56 ± 0.04 ^a^	0.56 ± 0.10 ^a^	0.51 ± 0.06 ^a^	0.59 ± 0.05 ^a^
Uterus (%)	0.26 ± 0.02 ^a^	0.28 ± 0.04 ^a^	0.25 ± 0.02 ^a^	0.30 ± 0.02 ^a^

Relative organ weights were calculated using maternal body weight before the removal of the gravid uterus. Means followed by the same letter within a row are not significantly different, as determined by a one-way ANOVA followed by Tukey’s post hoc test. The results are presented as the mean ± standard deviation (n = 5 per group). Relative organ weight was calculated as (organ weight/maternal body weight on the day of euthanasia) ×100.

**Table 4:** The effect of oral galactomannan administration on the absolute (g) and relative (%) organ weights of Wistar rats during the organogenesis phase (gestational days 6–15).

Organs	Control	Galactomannan		
		0.1 mg/kg	1 mg/kg	10 mg/kg
Liver (g)	12.89 ± 0.40 ^a^	12.71 ± 1.08 ^a^	12.59 ± 1.02 ^a^	11.75 ± 0.25 ^a^
Liver (%)	4.64 ± 0.14 ^a^	4.94 ± 0.50 ^a^	4.82 ± 0.21 ^a^	4.68 ± 0.07 ^a^
Kidney (g)	1.81 ± 0.19 ^a^	1.73 ± 0.07 ^a^	1.78 ± 0.18 ^a^	1.70 ± 0.22 ^a^
Kidney (%)	0.56 ± 0.15 ^a^	0.67 ± 0.01 ^a^	0.68 ± 0.08 ^a^	0.68 ± 0.09 ^a^
Spleen (g)	0.92 ± 0.36 ^a^	0.89 ± 0.30 ^a^	1.02 ± 0.53 ^a^	0.72 ± 0.14 ^a^
Spleen (%)	0.33 ± 0.11 ^a^	0.34 ± 0.11 ^a^	0.28 ± 0.13 ^a^	0.29 ± 0.06 ^a^
Heart (g)	0.93 ± 0.14 ^a^	0.87 ± 0.02 ^a^	0.82 ± 0.07 ^a^	0.80 ± 0.08 ^a^
Heart (%)	0.33 ± 0.04 ^a^	0.30 ± 0.02 ^a^	0.31 ± 0.03 ^a^	0.32 ± 0.02 ^a^
Lungs (g)	1.29 ± 0.28 ^a^	1.30 ± 0.14 ^a^	1.43 ± 0.21 ^a^	1.26 ± 0.22 ^a^
Lungs (%)	0.47 ± 0.08 ^a^	0.50 ± 0.03 ^a^	0.55 ± 0.07 ^a^	0.50 ± 0.07 ^a^
Thymus (g)	0.33 ± 0.04 ^a^	0.30 ± 0.04 ^a^	0.30 ± 0.08 ^a^	0.34 ± 0.03 ^a^
Thymus (%)	0.12 ± 0.01 ^a^	0.12 ± 0.01 ^a^	0.11 ± 0.03 ^a^	0.13 ± 0.01 ^a^
Ovary (g)	0.18 ± 0.06 ^a^	0.12 ± 0.02 ^a^	0.16 ± 0.03 ^a^	0.15 ± 0.02 ^a^
Ovary (%)	0.06 ± 0.02 ^a^	0.04 ± 0.00 ^a^	0.06 ± 0.01 ^a^	0.04 ± 0.01 ^a^

Relative organ weights were calculated using maternal body weight before the removal of the gravid uterus. Means followed by the same letter within a row are not significantly different, as determined by a one-way ANOVA followed by Tukey’s post hoc test. The results are presented as the mean ± standard deviation (n = 5 per group). Relative organ weight was calculated as (organ weight/maternal body weight on the day of euthanasia) ×100.

**Table 5:** Effects of oral administration of galactomannan on reproductive outcomes in Wistar rats treated during pre-implantation period (gestational days 1–6).

Reproductive Parameter	Control	0.1 mg/kg	1 mg/kg	10 mg/kg
Pregnant dams (n)	5	5	5	5
Corpora lutea/dam	10.40 ± 2.30	11.80 ± 1.92	10.20 ± 1.48	11.60 ± 2.97
Implantation sites/dam	10.00 ± 2.00	11.40 ± 1.14	9.60 ± 1.14	11.40 ± 3.36
Implantation index (%)	96.57 ± 4.80	97.33 ± 5.96	94.67 ± 7.67	97.14 ± 6.39
Pre-implantation loss (%)	3.43 ± 4.80	2.67 ± 5.96	5.33 ± 7.67	2.86 ± 6.39

Values are expressed as the mean ± standard deviation (SD). A pregnant dam was considered the experimental unit (n = 5 per group). The implantation index and pre-implantation loss were calculated separately for each dam before group-level summarization. No statistically significant differences were detected among groups (Kruskal–Wallis test, *p* > 0.05 for all parameters).

**Table 6:** The effect of oral galactomannan administration on the reproductive outcomes of Wistar rats exposed during the organogenesis period (gestational days 6–15).

Reproductive Parameters	Control		Galactomannan	
		0.1 mg/kg	1 mg/kg	10 mg/kg
Pregnant dams (n)	5	5	5	5
Live fetuses/litter	10.00 ± 1.73	9.40 ± 1.52	9.60 ± 2.41	8.80 ± 1.30
Dead fetuses/litter	0	0	0	0
Male fetuses/litter	4.60 ± 0.89	4.40 ± 1.34	4.60 ± 1.52	4.00 ± 1.00
Female fetuses/litter	5.40 ± 1.52	5.00 ± 1.22	5.00 ± 1.58	4.80 ± 1.79
Fetal body weight (g), litter mean	5.53 ± 0.26 ^ab^	5.30 ± 0.26 ^a^	5.70 ± 0.18 ^ab^	5.77 ± 0.17 ^b^
Sex ratio (M/F), litter-based	0.92 ± 0.33	0.93 ± 0.34	0.98 ± 0.37	0.99 ± 0.53
Implantations/litter	10.20 ± 1.30	9.60 ± 1.14	9.60 ± 2.41	9.20 ± 1.10
Resorption sites/litter	0.20 ± 0.45	0.20 ± 0.45	0.00 ± 0.00	0.40 ± 0.55
Corpora lutea/litter	10.20 ± 1.30	9.60 ± 1.14	9.80 ± 2.17	9.40 ± 1.34
Implantation index (%)	100.00 ± 0.00	100.00 ± 0.00	97.50 ± 5.59	98.18 ± 4.07
Post-implantation loss (%)	2.50 ± 5.59	2.50 ± 5.59	0.00 ± 0.00	4.50 ± 6.22
Absolute ovarian weight (g)	0.18 ± 0.06	0.12 ± 0.02	0.16 ± 0.03	0.15 ± 0.02
Relative ovarian weight (%)	0.06 ± 0.02	0.04 ± 0.00	0.06 ± 0.01	0.04 ± 0.01
Placental weight (g)	0.54 ± 0.08	0.55 ± 0.04	0.52 ± 0.02	0.53 ± 0.03
Placental index	0.10 ± 0.01	0.11 ± 0.01	0.10 ± 0.03	0.11 ± 0.10

Data are expressed as the mean ± standard deviation, with a pregnant dam/litter considered the experimental unit (n = 5 litters per group). Individual fetuses within the same litter were not considered independent experimental units. Fetal body weight represents the mean fetal weight calculated separately for each litter, with litter means used for group-level statistical analysis. The sex ratio and reproductive indices were likewise calculated for each litter before group-level analysis. Data were analyzed by a one-way ANOVA followed by Tukey’s multiple-comparison test when parametric assumptions were met or by the Kruskal–Wallis test followed by Dunn’s multiple-comparison test for nonparametric data. Statistical significance was set at *p* < 0.05. For fetal body weight, means sharing at least one lowercase superscript letter within the same row are not significantly different according to Tukey’s multiple-comparison test (*p* > 0.05), whereas means with no letters in common differ significantly (*p* < 0.05).

**Table 7:** The effect of oral galactomannan administration (0.1, 1.0, and 10 mg/kg) on skeletal alterations in the offspring of pregnant Wistar rats exposed during the organogenesis phase.

Skeletal Alterations	Control	Galactomannan		
		0.1 mg/kg	1 mg/kg	10 mg/kg
Incomplete ossification of proximal phalanges of the lower limbs	3/25 (12.0%); 3/5 L. (60%)	2/23 (8.7%); 2/5 L. (40%)	2/24 (8.3%); 2/5 L. (40%)	3/23 (13.0%); 2/5 L. (40%)
Incomplete ossification of proximal phalanges of the upper limbs	0/25 (0%); 0/5 L.	0/23 (0%); 0/5 L.	0/24 (0%); 0/5 L.	0/23 (0%); 0/5 L.
Cervical vertebrae agenesis	0/25 (0%); 0/5 L.	0/23 (0%); 0/5 L.	0/24 (0%); 0/5 L.	0/23 (0%); 0/5 L.
Caudal vertebrae agenesis	0/25 (0%); 0/5 L.	0/23 (0%); 0/5 L.	0/24 (0%); 0/5 L.	0/23 (0%); 0/5 L.
Incomplete ossification of sternebrae	2/25 (8.0%); 2/5 L. (40%)	1/23 (4.3%); 1/5 L. (20%)	1/24 (4.2%); 1/5 L. (20%)	2/23 (8.7%); 2/5 L. (40%)
Absence of xiphoid process	0/25 (0%); 0/5 L.	0/23 (0%); 0/5 L.	0/24 (0%); 0/5 L.	0/23 (0%); 0/5 L.
Enlarged fontanelle	0/25 (0%); 0/5 L.	0/23 (0%); 0/5 L.	0/24 (0%); 0/5 L.	0/23 (0%); 0/5 L.
Wavy ribs	0/25 (0%); 0/5 L.	0/23 (0%); 0/5 L.	0/24 (0%); 0/5 L.	0/23 (0%); 0/5 L.

Data are presented as fetal incidence [number of affected fetuses/number of fetuses examined (%)] followed by litter incidence [number of litters containing at least one affected fetus/number of litters examined (%)]. A pregnant dam/litter was considered the experimental unit (n = 5 litters per group), and individual fetuses within the same litter were not treated as independent experimental units. For the skeletal findings observed in at least one group, the proportion of affected fetuses was calculated separately for each litter and compared among groups using the Kruskal–Wallis test. No statistical comparison was performed for findings with zero incidence in all groups. Statistical significance was set at *p* < 0.05. L.: litters.

## Data Availability

The data presented in this study are available on request from the corresponding author.

## Supplementary Materials

The following supporting information can be downloaded at https://www.mdpi.com/article/10.3390/gels12100936/s1, Figure S1: Fourier-transform infrared (FTIR) spectrum of galactomannan (GAL) extracted from *Cassia grandis* seeds.

## Abbreviations

The following abbreviations are used in this manuscript:

ANOVA	Analysis of Variance
DMEM	Dulbecco’s Modified Eagle Medium
DMSO	Dimethyl Sulfoxide
FTIR	Fourier-Transform Infrared Spectroscopy
GD	Gestational Day
IS	Implantation Site
OECD	Organization for Economic Co-operation and Development
MTT	[3-(4,5-Dimethylthiazol-2-yl)-2,5-Diphenyl Tetrazolium Bromide]
NOAEL	No-Observed-Adverse-Effect Level
OECD	Organisation for Economic Co-operation and Development

## References

[1] Suhas P., Mahesh B., Divakara S.G., Nanjundaswamy G.S., Mahadeva Prasad C.S., Sionkowska A., Popat K.C., Channe Gowda D. (2025). Synergistic approaches in natural and synthetic polymer blends for biomedical applications—A review. Eur. Polym. J..

[2] Albuquerque P.B.S., de Oliveira W.F., dos Santos Silva P.M., Correia M.T.S., Kennedy J.F., Coelho L.C.B.B. (2022). Skincare application of medicinal plant polysaccharides—A review. Carbohydr. Polym..

[3] Liu Z.H., Niu F.J., Xie Y.X., Xie S.M., Liu Y.N., Yang Y.Y., Wan X.H. (2020). A review: Natural polysaccharides from medicinal plants and microorganisms and their anti-herpetic mechanism. Biomed. Pharmacother..

[4] Leal M.R.S., Albuquerque P.B.S., Rodrigues N.E.R., Oliveira P.M.S., Oliveira W.F., Correia M.T.S., Kennedy J.F., Coelho L.C.B.B. (2024). A review on the use of polysaccharides as thickeners in yogurts. Carbohydr. Polym. Technol. Appl..

[5] (1992). Wealth of India-Raw Materials. The Wealth of India: Raw Materials.

[6] Joshi H., Kapoor V.P. (2003). *Cassia grandis* Linn. f. seed galactomannan: Structural and crystallographical studies. Carbohydr. Res..

[7] Albuquerque P.B.S., Barros W., Santos G.R.C., Correia M.T.S., Mourão P.A.S., Teixeira J.A., Carneiro-da-Cunha M.G. (2014). Characterization and rheological study of the galactomannan extracted from seeds of *Cassia grandis*. Carbohydr. Polym..

[8] Albuquerque P.B.S., Silva C.S., Soares P.A.G., Barros W., Correia M.T.S., Coelho L.C.B.B., Teixeira J.A., Carneiro-da-Cunha M.G. (2016). Investigating a galactomannan gel obtained from *Cassia grandis* seeds as immobilizing matrix for Cramoll lectin. Int. J. Biol. Macromol..

[9] Albuquerque P.B.S., Cerqueira M.A., Vicente A.A., Teixeira J.A., Carneiro-da-Cunha M.G. (2017). Immobilization of bioactive compounds in *Cassia grandis* galactomannan-based films: Influence on physicochemical properties. Int. J. Biol. Macromol..

[10] Albuquerque P.B.S., Soares P.A.G., Aragão-Neto A.C., Albuquerque G.S., Silva L.C.N., Lima-Ribeiro M.H.M., Silva Neto J.C., Coelho L.C.B.B., Correia M.T.S., Teixeira J.A. (2017). Healing activity evaluation of the galactomannan film obtained from *Cassia grandis* seeds with immobilized *Cratylia mollis* seed lectin. Int. J. Biol. Macromol..

[11] Souza A.A., Ribeiro K.A., Seixas J.R.P.C., Silva Neto J.C., Santiago M.G.P.F., Aragão-Neto A.C., Lima-Ribeiro M.H.M., Borba E.F.O., Silva T.G., Kennedy J.F. (2023). Effects including photobiomodulation of galactomannan gel from *Cassia grandis* seeds in the healing process of second-degree burns. Int. J. Biol. Macromol..

[12] Barbosa J.M.A.X., Nunes D.M., Sabiá G.A., Soares A.F., Silva T.G.D., Lima L.R.A.D. (2024). Gastroprotective activity of the galactomannan extracted from *Cassia grandis* seeds: An in vivo study. BME Horiz..

[13] Duarte C.A.L., da Silva M.G., Porto A.L.F., de Albuquerque Wanderley M.C., da Silva S.S.S., de Andrade A.F., Bezerra R.P., Converti A., Ramos D.G., de Araújo Viana Marques D. (2024). Application of the galactomannan gel from *Cassia grandis* seeds for biomedical purposes: Study of the incorporation of collagenases and their release profile. Carbohydr. Polym..

[14] Lu Z., Guo Y., Xu D., Xiao H., Dai Y., Liu K., Chen L., Wang H. (2023). Developmental toxicity and programming alterations of multiple organs in offspring induced by medication during pregnancy. Acta Pharm. Sin. B.

[15] Hajar A., Abdelmounaima B., Hamid K., Jaouad L., Abdelfattah A., Majdaoui B., Loubna E.Y., Mohammed L., Rachidac A., Abderrahman C. (2024). Developmental toxicity of *Moringa oleifera* and its effect on postpartum depression, maternal behavior and lactation. S. Afr. J. Bot..

[16] Rodrigues N.E.R., Oliveira A.R.d.S., Lima S.M.d.A., Nunes D.M., Albuquerque P.B.S.d., da Cunha M.d.G.C., Wanderley A.G., Júnior F.M.R.d.S., Silva J.B.N.F., Teixeira Á.A.C. (2023). Effect of the aqueous extract of *Chrysobalanus icaco* leaves on maternal reproductive outcomes and fetal development in Wistar rats. Curr. Issues Mol. Biol..

[17] Souza M.R., Brito E.C.B., Furtado L.S., Barco V.S., Cruz L.L.D., Moraes-Souza R.Q., Monteiro G.C., Lima G.P.P., Damasceno D.C., Volpato G.T. (2023). Maternal-fetal toxicity of *Strychnos pseudoquina* extract treatment during pregnancy. J. Ethnopharmacol..

[18] Moura Neto E., Sombra V.G., Richter A.R., Abreu C.M.W.S., Maciel J.S., Cunha P.L.R., Ono L., Sierakowski M.R., Feitosa J.P.A., de Paula R.C.M. (2014). Chemically sulfated galactomannan from *Dimorphandra gardneriana* seed: Characterization and toxicity evaluation. Carbohydr. Polym..

[19] Chrestani F., Sierakowski M.R., de Andrade Uchoa D.E., Nozawa C., Sassaki G.L., Gorin P.A., Ono L. (2009). In vitro antiherpetic and antirotaviral activities of a sulfate prepared from *Mimosa scabrella* galactomannan. Int. J. Biol. Macromol..

[20] Ganter J.L.M.S., Cardoso A.T.M., Kaminski M., Reicher F. (1997). Galactomannan from the seeds of *Mimosa scabrella*: A scale-up process. Int. J. Biol. Macromol..

[21] Marques F.C.J., Nascimento F.G.S., Nonato D.T.T., Assreuy A.M.S., Chaves E.M.C., Aragão G.F., Soares P.M.G., Castro R.R. (2025). Acute toxicity study of bioactive galactomannans from seeds of two nontraditional leguminosae. Rev. Caatinga.

[22] Pascoal K.L.L., Siqueira S.M.C., Amorim A.F.V., Ricardo N.M.P.S., Menezes J.E.S.A., Silva L.C., Araújo T.G., Almeida-Neto F.W.Q., Marinho E.S., Morais S.M. (2021). Physical-chemical characterization, controlled release, and toxicological potential of galactomannan-bixin microparticles. J. Mol. Struct..

[23] Leal-Silva T., Souza M.R., Cruz L.L., Moraes-Souza R.Q., Paula V.G., Soares T.S., Dela Justina V., Giachini F.R., Damasceno D.C., Américo M.F. (2023). Toxicological effects of *Morinda citrifolia* L. fruit extract on maternal reproduction and fetal development in rats. Drug Chem. Toxicol..

[24] Chahoud I., Ligensa A., Dietzel L., Faqi A.S. (1999). Correlation between maternal toxicity and embryo/fetal effects. Reprod. Toxicol..

[25] Ezeja M.I., Anaga A.O., Asuzu I.U. (2014). Acute and sub-chronic toxicity profile of methanol leaf extract of *Gouania longipetala* in rats. J. Ethnopharmacol..

[26] Deshpande P., Mohan V., Thakurdesai P. (2016). Preclinical safety evaluation of low molecular weight galactomannans based standardized fenugreek seeds extract. EXCLI J..

[27] Qin G., Gao Y., Wen P., Liang G., Zhao P., Dong B., Tang S., Shekh K. (2022). Evaluation of the genotoxicity and teratogenicity of xylan using different model approaches. Drug Chem. Toxicol..

[28] Wise L.D., DeSesso J.M. (2024). A critical look at adjusted fetal weights in rats. Birth Defects Res..

[29] Lemonica I.P., Damasceno D.C., Di-Stasi L.C. (1996). Study of the embryotoxic effects of an extract of rosemary (*Rosmarinus officinalis* L.). Braz. J. Med. Biol. Res..

[30] Melo I.M.F., Silva W.E., Teixeira A.A.C., Wanderley-Teixeira V., Barreto S.B.F.P., Lapa Neto C.J.C., Andrade F.M. (2014). Effect of enrofloxacin on the blastocyst endometrial interactions and their impact on placental and fetal development in rats. Arq. Bras. Med. Vet. Zootec..

[31] Medeiros M.A., Riet-Correa F., Dantas F.P.M., Santos J.R.S., Medeiros R.M.T. (2014). Teratogenic effects of *Prosopis juliflora* in rats and analysis of the toxicity of the pods. Pesqui. Vet. Bras..

[32] Bernardi M.M., Spinosa H.S., Spinosa H.S., Górniak S.L., Bernardi M.M. (2017). Exposição aos medicamentos durante o período perinatal. Farmacologia Aplicada à Medicina Veterinária.

[33] Meguem S.O., Lienou L.L., Goka M.S.C., Tagne R.S., Yemele D.M., Telefo P.B. (2021). Effects of the aqueous extract of *Dicliptera verticillata* on fertility and different stages of gestation in female rats. Zygote.

[34] Almeida F.C.G., Lemonica I.P. (2000). The toxic effects of *Coleus barbatus* B. on the different periods of pregnancy in rats. J. Ethnopharmacol..

[35] Kalo D., Yaacobi-Artzi S., Manovich S., Michaelov A., Komsky-Elbaz A., Roth Z. (2024). Environmental stress-induced alterations in embryo developmental morphokinetics. J. Xenobiotics.

[36] OECD (2025). Test No. 421: Reproduction/Developmental Toxicity Screening Test. OECD Guidelines for the Testing of Chemicals, Section 4.

[37] Ortega M.A., Fraile-Martínez O., García-Montero C., Sáez M.A., Álvarez-Mon M.A., Torres-Carranza D., Álvarez-Mon M., Bujan J., García-Honduvilla N., Bravo C. (2022). The pivotal role of the placenta in normal and pathological pregnancies: A focus on preeclampsia, fetal growth restriction, and maternal chronic venous disease. Cells.

[38] Londero A.P., Bertozzi S., Visentin S., Fruscalzo A., Driul L., Marchesoni D. (2013). High placental index and poor pregnancy outcomes: A retrospective study of 18 386 pregnancies. Gynecol. Endocrinol..

[39] Weidu H. (2006). Shanghai Institute for Preventive Medicine Testing Report: Summary of One and Two Stage Toxicological Tests on RheoRanger SR.

[40] Collins T.F., Welsh J.J., Black T.N., Graham S.L., O’Donnell M.W. (1987). Study of the teratogenic potential of guar gum. Food Chem. Toxicol..

[41] Borzelleca J.F., Ladu B.N., Senti F.R., Egle J.L. (1993). Evaluation of the safety of tara gum as a food ingredient: A review of the literature. J. Am. Coll. Toxicol..

[42] Chamorro-Cevallos G., Mojica-Villegas M.A., García-Martínez Y., Pérez-Gutiérrez S., Madrigal-Santillán E., Vargas-Mendoza N., Cristóbal-Luna J.M. (2022). A complete review of Mexican plants with teratogenic effects. Plants.

[43] Posobiec L.M., Kopp C., Murzyn S.M., Olitan T., Rendemonti J., French J., Tamborini E., Campey J., Longo M., Danberry T. (2023). Harmonization of criteria and terminology in fetal rat skeletal evaluations. Birth Defects Res..

[44] Paumgartten F., Solecki R., Buschmann J., Clark R., Grote K., Rauch M., Chahoud I. (2009). Harmonization of terminology in developmental toxicology: The quest for a more precise description and a harmonized classification of fetal observations. Reprod. Toxicol..

[45] Fritz H., Hess R. (1970). Ossification of the rat and mouse skeleton in the perinatal period. Teratology.

[46] Carney E.W., Kimmel C.A. (2007). Interpretation of Skeletal Variations for Human Risk Assessment: Delayed Ossification and Wavy Ribs. Birth Defects Res. Part B Dev. Reprod. Toxicol..

[47] Musa A.H., Gebru G., Debella A., Makonnen E., Asefa M., Woldekidan S., Abebe A., Lengiso B., Bashea C. (2022). Prenatal developmental toxicity study of herbal tea of *Moringa stenopetala* and *Mentha spicata* leaves formulation in Wistar rats. Toxicol. Rep..

[48] Chahoud I., Paumgartten F.J.R. (2005). Relationships between fetal body weight of Wistar rats at term and the extent of skeletal ossification. Braz. J. Med. Biol. Res..

[49] Aliverti V., Bonanomi L., Giavini E., Leone V.G., Mariani L. (1979). The extent of fetal ossification as an index of delayed development in teratogenic studies on the rat. Teratology.

[50] Ariyuki F., Ishihara H., Higaki K., Yasuda M. (1982). A study of fetal growth retardation in teratological tests: Relationship between body weight and ossification of the skeleton in rat fetuses. Teratology.

[51] Marques F.C.J., Pantoja P.S., Matos V.E.A., Silva R.O., Damasceno S.R.B., Franco Á.X., Alves R.C., Justino P.F.C., Souza M.H.L.P., Feitosa J.P.A. (2019). Galactomannan from the seeds of *Caesalpinia pulcherrima* prevents indomethacin-induced gastrointestinal damage via neutrophil migration. Int. J. Biol. Macromol..

[52] Dallazen J.L., Ciapparini P.G., Maria-Ferreira D., da Luz B.B., Klosterhoff R.R., Felipe L.P.G., Silva B.J.G., Cordeiro L.M.C., Werner M.F.P. (2024). Arabinan-rich pectic polysaccharide fraction from *Malpighia emarginata* fruits alleviates inflammatory pain in mice. Food Res. Int..

[53] Mosmann T. (1983). Rapid colorimetric assay for cellular growth and survival: Application to proliferation and cytotoxicity assays. J. Immunol. Methods.

[54] Cooper R.L., Goldman J.M., Vandenbergh J.G., Chapin R.E., Heindel J. (1993). Monitoring of estrous cycle in the laboratory rodent by vaginal lavage. Methods in Toxicology.

[55] Wilson J.G., Wilson J.G., Warkany J. (1965). Methods for administering agents and detecting malformations in experimental animals. Teratology: Principles and Techniques.

[56] Manson J.M., Zenick H., Costlow R.D., Hayes A.W. (1982). Teratology test methods for laboratory animals. Principles and Methods of Toxicology.

[57] Staples R.E., Schnell V.L. (1964). Refinements in rapid clearing technique in the KOH-alizarin red S method for fetal bone. Stain. Technol..

[58] Makris S.L., Solomon H.M., Clark R., Shiota K., Barbellion S., Buschmann J., Ema M., Fujiwara M., Grote K., Hazelden K.P. (2009). Terminology of developmental abnormalities in common laboratory mammals (Version 2). Congenit. Anom..

