# Supplementary material for: Maternal, Embryofetal and Teratogenic Toxicity of Gel-Forming Galactomannan from Cassia grandis in Wistar Rats
Source: Gels. 2026 Oct 18;12(10):936. doi: 10.3390/gels12100936 (PMC13704428; doi:10.3390/gels12100936)
Supplement: Supplementary file 1 [file gels-12-00936-s001.zip › gels-4612121-supplementary.pdf]

Supplementary file

Figure S1. Fourier Transformed Infrared (FTIR) spectroscopy of the galactomannan (GAL) extracted from *Cassia grandis* seeds.

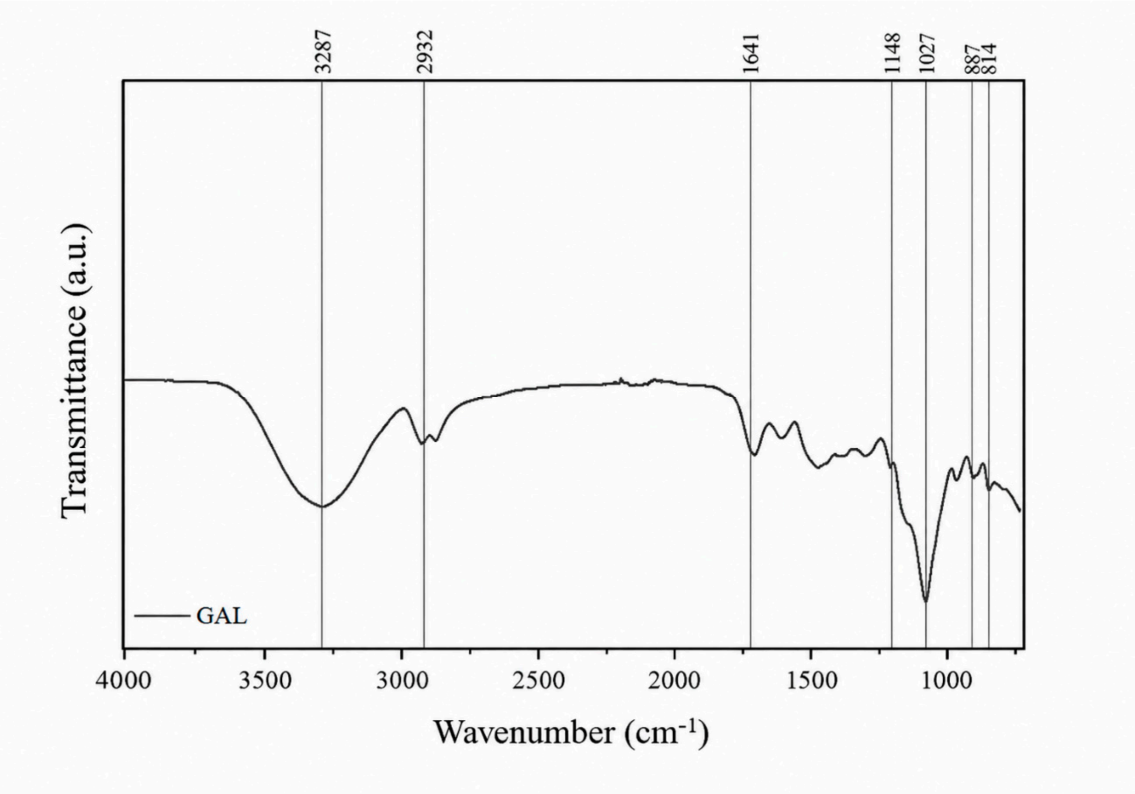

The FTIR spectrum confirms that the extracted material is a galactomannan. Absorption peak at 3287 cm^{-1} is characteristic of a carbohydrate ring and represents hydroxyl stretching vibration of polysaccharides and water involved in hydrogen bonding. The absorption at 2932 cm^{-1} is assigned to the stretching vibration of the methylene group (C − H). In the anomeric region (950 − 700 cm^{-1}), the polysaccharide exhibited the obvious characteristic absorption at 887 and 814 cm^{-1}, assigned to α-d-galactopyranose and β-d-mannopyranose, respectively. At 1148 cm^{-1}, C-O-C stretching vibration is characteristic in glycosidic bonds, thus indicating that the extraction protocol was efficient in preserving the polysaccharide backbone. Finally, absorption at 1027 cm $^{-1}$ represents the carbohydrate fingerprint region, and 1641 cm^{-1} is characteristic of H-O-H bending and mannose/galactose ring vibrations.
